# Increased Circulation and Adipose Tissue Levels of DNAJC27/RBJ in Obesity and Type 2-Diabetes

**DOI:** 10.3389/fendo.2018.00423

**Published:** 2018-08-07

**Authors:** Preethi T. Cherian, Irina Al-Khairi, Devarajan Sriraman, Ahmad Al-Enezi, Dalal Al-Sultan, Mohammed AlOtaibi, Saad Al-Enezi, Jaakko Tuomilehto, Fahd Al-Mulla, Jehad A. Abubaker, Mohamed Abu-Farha

**Affiliations:** ^1^Biochemistry and Molecular Biology Unit, Dasman Diabetes Institute, Kuwait City, Kuwait; ^2^National Dasman Diabetes Biobank, Dasman Diabetes Institute, Kuwait City, Kuwait; ^3^Functional Genomic Unit, Dasman Diabetes Institute, Kuwait City, Kuwait; ^4^Research Division, Dasman Diabetes Institute, Kuwait City, Kuwait

**Keywords:** DNAJC27, heat shock proteins, heat shock response, obesity, type 2 diabetes

## Abstract

Heat shock response is an essential cellular stress response. Dysregulation of various heat shock proteins (HSPs), within the heat shock response (HSR) pathway, play a vital role in this host-defense mechanism contributing to obesity-induced insulin resistance and type 2 diabetes (T2D). Previously, we have reported changes in the expression levels of several HSPs such as HSP40, HSP60, HSP70, and HSP90 in obese compared with lean individuals. DNAJC27 is a member of the HSP40 protein family that was previously identified as a body mass index (BMI) associated locus in genome-wide association (GWAS) studies. However, not much is known about the changes in DNAJC27 expression levels in obesity and T2D. In the present study, we aimed at understanding changes in DNAJC27 expression levels in plasma, peripheral blood mononuclear cells (PBMCs) and adipose tissue in association with obesity and T2D. A total of 277 individuals enrolled including 160 non-diabetic (96 non-obese and 64 obese) and 117 T2D (45 non-obese and 72 obese) individuals. Plasma level of DNAJC27 was significantly higher in obese individuals (6.28 ± 0.64 ng/mL) compared with non-obese individuals (4.8 ± 0.45 ng/mL) with *P* = 0.043. Dividing the population based on diabetes status showed that there was a significant increase in the plasma level of DNAJC27 in obese (6.90 ± 1.3 ng/mL) compared with non-obese individuals (3.81 ± 0.43 ng/mL) (*P* = *0.033*) in the non-diabetic group. Similarly, DNAJC27 expression level was also higher in PBMCs and adipose tissue of obese individuals. DNAJC27 was found to be associated with leptin and resistin, adipokines known to be dysregulated in obesity, that stimulate inflammatory processes leading to metabolic disorders. In conclusion, our data show that DNAJC27 is elevated in obese and T2D individuals and was positively associated with obesity biomarkers such as leptin and resistin suggesting that this protein may play a role in the pathophysiology of these disorders.

## Introduction

Obesity is a global epidemic that is associated with numerous comorbidities such as T2D and cardiovascular disease (1). Chronic low-grade inflammation and altered stress response are associated with obesity and play a key role in the pathology of insulin resistance and T2D (2–5). Hotamisligil et al. identified TNF-α as the first molecular link between obesity and inflammation (6). This inflammatory cytokine was observed to be over-expressed in the adipose tissue and in the muscles of animal models of obesity and in humans (7–9). Several other pro-inflammatory cytokines such as Interleukin-1β (IL1β), Monocyte Chemoattractant Protein-1 (MCP1), C-reactive protein, and Interferon-γ (IFN-γ) were also shown to be dysregulated in individuals with obesity. Studies have reported that the increased production of these inflammatory cytokines precedes increased inflammation, resulting in obesity-induced insulin resistance (10, 11). Additionally, other inflammatory mediators such as the adipokines leptin, resistin, adiponectin, and visfatin have been shown to be dysregulated in adipocytes derived from obese individuals (9, 12). Imbalance in the expression and secretion of these molecules and other cytokines affect insulin resistance and eventually lead to T2D. Understanding the molecular mechanisms involved in chronic low-grade inflammation is essential for developing strategies to mitigate obesity and disorders associated with it.

Obesity is known to alter stress response pathways such as those involved in oxidative stress, endoplasmic reticulum (ER) stress, and heat shock response (HSR) (3, 13). Inflammatory and stress response pathways are closely interconnected because they trigger the activation of various stress kinases such as c-Jun NH2 terminal kinase (JNK), inhibitor of κB kinase (IKK), and protein kinase C (PKC) (5, 14, 15). These kinases are involved in insulin signaling through the phosphorylation of insulin receptor substrates (IRS-1 and IRS-2) on serine and threonine residues. Dysregulation of these kinases due to obesity disrupts the interaction between IRS and insulin receptor (IR), which leads to impairment in insulin signaling (16). This phenomenon may provide an explanation for obesity-induced insulin resistance (2, 4, 5, 14).

The HSR pathway is one of the major stress response pathways that regulate cellular stress kinases. Obesity leads to an imbalance in the HSR pathway. HSPs are highly conserved molecular chaperones that play a crucial role in this pathway by maintaining cellular homeostasis (17). For example, HSP72 has been shown to improve insulin sensitivity and reduce inflammation. It has been observed that in T2D patients, HSP72 expression level was lower than that in their non-diabetic counterparts (15, 18). We have studied DNAJB3, another heat shock protein (HSP) that belongs to the HSP40 protein family and observed it to be downregulated in obese individuals and restored following physical exercise (14). Similarly, DNAJB3 expression was reduced in obese T2D individuals as compared with obese non-diabetic individuals (19). Through proteomic analysis of PBMCs comparing obese and non-obese individuals, we found that the expression of DNAJC27, another member of the HSP40 protein family, was increased in obese individuals (20). DNAJC27 has also been identified by GWAS to be associated with BMI in East Asians (21). However, little is known about the expression and role of this protein in obesity and T2D. In this study, we investigated the expression levels of DNAJC27 in circulation, PBMCs and adipose tissue. In addition, we discuss its correlation with other biochemical markers associated with obesity and T2D to understand its clinical significance.

## Materials and methods

### Study population

This study was conducted on adult obese (BMI > 30 kg/m^2^) and non-obese (20 ≤ BMI ≥ 30 kg/m^2^) male and female individuals. A total of 277 participants comprising 160 non-diabetic (96 non-obese and 64 obese) and 117 T2D (45 non-obese and 72 obese) individuals were enrolled in this study. Diabetes was defined by fasting plasma glucose ≥7 mmol/l, under treatment, or self-reporting of previously diagnosed T2D (22, 23). The T2D patients included in our study were on anti-diabetic medication which were mainly Metformin or its analogs, GLP-1 receptor agonists, or Insulin (Lantus). Written informed consent was obtained from all participants prior to their participation. The study was approved by the ethical review board of Dasman Diabetes Institute and has been carried out in accordance with the guidelines present in the ethical declaration of Helsinki. Participants who were morbidly obese (BMI > 40 kg/m^2^), had prior major illness, and/or were under medication or supplements known to have an influence on body composition or bone mass were excluded from the study (14).

### Blood and tissue sampling

Venous blood samples were collected using vacutainer EDTA tubes after a minimum of 8-h fasting. The blood was centrifuged at 400 × g for 10 min at room temperature. Plasma was separated, then aliquoted and stored at −80°C until the assay was performed. PBMCs and subcutaneous adipose tissue (SAT) biopsies were obtained as described (14, 20). The biopsy samples were immediately rinsed in cold PBS, divided into four pieces each, and appropriately stored until the assay was performed.

### Anthropometric measurements and blood biochemistry

Anthropometric measurements of height and weight were obtained for calculating BMI using weight (kg) to height squared (m) ratio. Fasting blood glucose (FBG) and lipid profiles including Triglyceride (TGL), low density lipoprotein (LDL), high density lipoprotein (HDL) as well as total Cholesterol (TC) were measured using a Siemens Dimension RXL chemistry analyser (Diamond Diagnostics, Holliston, MA). HbA1c levels were determined using the Variant™ device (Bio-Rad, Hercules, CA). Plasma levels of inflammatory and metabolic markers were measured using bead-based multiplexing technology. Median fluorescence intensities were measured using a Bioplex200 system and analyzed using the software Bio-plex Manager version 6 (Bio-Rad, Hercules, CA). All the above assays were carried out in accordance with the manufacturer's instructions. Insulin resistance was calculated using HOMA-IR formula: FBG (mmol/L) × fasting insulin (mU/L) / 22.5 (23).

### RNA extraction and gene expression via quantitative real-time PCR

Total RNA was extracted from PBMCs using the All Prep RNA/DNA/Protein kit (Qiagen Inc. Valencia, CA). SATs to be used for mRNA analysis were immediately stored after biopsy in 1 ml RNAlater RNA Stabilization Reagent (Qiagen Inc.) at −80°C. Total RNA was extracted from the SAT using RNeasy Lipid Tissue Mini Kit (Qiagen Inc., Valencia, CA). RNA samples obtained from PBMCs and SAT were quantified for assessing their quality and concentration using Epoch microplate spectrophotometer (BioTek Instruments, Inc.). A 1-μg aliquot of each extracted RNA sample was reverse transcribed for preparing cDNA using High Capacity cDNA Reverse Transcription Kits (Applied Bio systems, Foster City, CA). Quantitative real-time polymerase chain reaction (qRT-PCR) was carried out using the Rotor-Disc 100 system with SYBR Green, normalizing test sample values to those of GAPDH (Qiagen Inc. Valencia CA). The primers used for DNAJC27 were:

DNAJC27-forward 5′-TGCACATCAGCAGAGGAAAG-3′ and DNAJC27-reverse 5′-GAAGAGGCCAACTTTGCTGA-3′. Primers used for GAPDH were GAPDH-forward 5′-AACTTTGGCATTGTGGAAGG-3′ and GAPDH-reverse 5′-TGTGAGGGAGATGCTCAGTG-3′. Relative expression was assessed using the ΔΔCT method (24).

### Western blot analysis

Western blot analysis was performed using whole PBMC extracts prepared in RIPA buffer (50 mM Tris HCL pH 7.5, 150 mM NaCl, 1% Triton × 100, 1 mM EDTA, 0.5% Sodium deoxycholate, and 0.1% SDS). Protein concentration was determined using the Bradford method with bovine serum albumin (sigma Aldrich) dissolved in RIPA as a standard. The protein samples (20 μg) were resolved in 10% acrylamide gels, then transferred onto PVDF membranes, and blocked with 5% non-fat dried milk in Tris-buffered saline containing 0.05% Tween 20 for 2 h at room temperature. The membranes were incubated with primary antibody against DNAJC27 diluted 1:1000 (Abgent Cat. # AP12755a, San Diego, California, USA) overnight at 4°C. The next day, the membranes were washed and incubated with secondary anti-rabbit antibody (GE Amersham Cat. # NA9340) with a 1:10,000 dilution). Protein bands were visualized using chemiluminescence (Super signal, Thermo Fischer) and images were captured using Versadoc 5000 system (Bio-rad, Hercules, CA).

### Measurement of plasma levels of DNAJC27 using ELISA

Plasma level of DNAJC27 was measured using an ELISA kit (Wuhan EIAab Science Co., Wuhan, China). Plasma samples were thawed on ice and then centrifuged for 5 min at 10,000 × g at 4°C to remove cells or platelets remaining within the sample (14, 19). Samples were diluted 4x with sample diluent. ELISA was performed in accordance to kit instructions; briefly, the samples and standards were loaded onto the assay plate and incubated for 2 h at 37°C. Next, the samples were washed and incubated for 1 h at 37°C, successively following the addition of conjugated antibody and then streptavidin. Lastly, the plate was incubated with the substrate TMB for 30 min at 37°C, the reaction was stopped using acidic stop solution and the absorbance was measured using a Synergy H4 plate reader at a wavelength of 450 nm. All reagents used were provided in the kit. Intra-assay coefficient of variation was 3.0–5.0%, while the inter-assay coefficient of variation was 3.5–6.0%.

### Statistics

Statistical analysis was performed on the 277 participants, who were classified as 141 obese (BMI > 30 kg/m^2^) and 136 non-obese (20 ≤ BMI ≥ 30 kg/m^2^) and Student's *t*-test was used for the comparison of various clinical and biochemical parameters tested. In order to assess the differences between the non-diabetic (non-obese and obese) and diabetic (non-obese and obese) individuals, a two-way ANOVA with *post-hoc* Bonferroni test was performed on the whole population (*n* = 277). Spearman's rho (*r*) correlation coefficient was used to determine the association of DNAJC27 with obesity and diabetes biomarkers including leptin, resistin, adiponectin, and HOMA-IR. All data were reported as mean ± standard error of mean (SEM). Statistical assessment was two-sided and considered statistically significant at *P* < *0.05*. All analyses were performed using SAS software (version 9r; SAS Institute).

## Results

### Characteristics of the population under study

Selected characteristics of our sample population stratified by BMI are presented in Table 1. Age, HDL, FBG, and HbA1C were significantly higher in obese than in non-obese individuals (*P* < 0.05). After classifying the population based on diabetes, within the non-diabetic population, a significantly higher FBG, and TGL (*P* < 0.05) was seen in the obese individuals when compared to non-obese individuals (Table 2). Among the people with T2D, it was observed that FBG and HbA1C were significantly higher in obese than non-obese people (Table 2). A two-way ANOVA was conducted that examined the effect of obesity and diabetes on FBG, HBA1C and PAI-1. There was a statistically significant interaction between the effects of obesity and diabetes on FBG (*P* = 0.010); HBA1C (*P* < 0.001); PAI-1 (*P* = 0.013).A two-way ANOVA was conducted that examined the effect of obesity and diabetes on DNAJC27. There was no statistically significant interaction between the effects of obesity and diabetes (*P* = 0.281) (Table 2). The whole population was also classified based on T2D and the results are provided in Supplementary Table 1.

**Table 1:** Physical and biochemical characteristics of the whole study population categorized based on obesity.

**All population (*n* = 277)**	**Non-obese (*n* = 141)**	**Obese (*n* = 136)**	***P-*value**
Age (Years)	44.21 ± 1.05	48.30 ± 1.03	**0.006**
TC (mmol/L)	5.03 ± 0.10	5.05 ± 0.09	0.851
HDL (mmol/L)	1.34 ± 0.04	1.21 ± 0.03	**0.019**
LDL (mmol/L)	3.12 ± 0.09	3.16 ± 0.08	0.791
TGL (mmol/L)	1.29 ± 0.09	1.52 ± 0.09	0.057
FBG (mmol/L)	5.96 ± 0.17	7.3 ± 0.27	**<0.0001**
HbA1c (%)	6.06 ± 0.12	7.05 ± 0.16	**<0.0001**
Insulin (U/L)	17.33 ± 1.82	28.20 ± 3.23	**0.004**
C-peptide (pg/ml)	3.90 ± 0.54	4.25 ± 0.48	0.625
Leptin (ng/ml)	5.43 ± 0.36	9.61 ± 0.62	**<0.0001**
Resistin(ng/ml)	3.31 ± 0.14	3.40 ± 0.16	0.682
PAI-1 (ng/ml)	14.64 ± 0.63	15.68 ± 0.60	0.236
Visfatin (ng/ml)	3.99 ± 0.31	4.32 ± 0.24	0.408
Adiponectin (μg/ml)	5.09 ± 0.36	4.13 ± 0.24	0.027

*Data are presented as mean ± SEM. Student's T-test was used for the comparison of various clinical and biochemical parameters tested. n = 277. TC, Total Cholesterol; HDL, high density lipoprotein; LDL, low density lipoprotein; and TGL, triglyceride level; FBG, fasting blood glucose; HbA1c, hemoglobin A1c; PAI-1, Plasminogen activator inhibitor-1. Bold values represent significance*.

**Table 2:** Physical and Biochemical characteristics of the non-diabetic and diabetic population categorized based on obesity.

	**Non-diabetic (*n* = 160)**		**Diabetic (*n* = 117)**		***P-*value**	***Post-hoc* Bonferroni *p*-value**
	**Non-Obese (*n* = 96)**	**Obese (*n* = 64)**	**Non-Obese (*n* = 45)**	**Obese (*n* = 72)**	**A vs. B** **C vs. D** **ANOVA**	**A vs. C** **A vs. D** **B vs. C** **B vs. D**
	**(A)**	**(B)**	**(C)**	**(D)**		
Age (in years)	40.63 ± 1.23	43.72 ± 1.65	51.87 ± 1.40	52.38 ± 1.10	0.139 0.799 < **0.001**	<**0.001** < **0.001** **0.002** < **0.001**
Total cholesterol (mmol/L)	5.13 ± 0.10	5.13 ± 0.11	4.82 ± 0.23	5.00 ± 0.13	0.831 0.190 0.383	0.754 1.000 0.870 1.000
HDL (mmol/L)	1.38 ± 0.05	1.27 ± 0.05	1.25 ± 0.09	1.14 ± 0.04	0.291 0.673 **0.013**	0.744 **0.006** 1.000 0.521
LDL (mmol/L)	3.21 ± 0.10	3.28 ± 0.11	3.00 ± 0.18	3.04 ± 0.13	0.608 0.356 0.271	1.000 1.000 0.568 0.987
TGL (mmol/L)	1.17 ± 0.11	1.31 ± 0.09	1.51 ± 0.16	1.72 ± 0.14	**0.022** 0.193 **0.006**	0.407 **0.004** 1.000 0.103
FBG (mmol/L)	5.27 ± 0.15	5.46 ± 0.12	7.30 ± 0.34	8.93 ± 0.40	**0.019** **0.008** < **0.001**	<**0.001** < **0.001** < **0.001** **0.001**
HBA1C %	5.64 ± 0.11	5.62 ± 0.07	6.85 ± 0.22	8.32 ± 0.21	0.293 < **0.001** < **0.001**	<**0.001** < **0.001** < **0.001** < **0.001**
Insulin (U/L)	15.86 ± 2.30	30.82 ± 4.81	20.36 ± 2.95	25.81 ± 4.37	**0.001** 0.763 **0.022**	1.000 0.263 0.517 1.000
C-Peptide (pg/ml)	4.42 ± 0.76	4.67 ± 0.68	3.00 ± 0.65	3.95 ± 0.67	0.533 0.142 0.485	1.000 1.000 0.892 1.000
Leptin (ng/ml)	5.31 ± 0.44	10.11 ± 0.95	5.68 ± 0.65	9.03 ± 0.77	<**0.001** **0.004** < **0.001**	1.000 **0.001** **0.001** 1.000
Resistin (ng/ml)	3.38 ± 0.19	3.38 ± 0.25	3.16 ± 0.16	3.41 ± 0.20	0.679 0.581 0.881	1.000 1.000 1.000 1.000
PAI-1 (ng/ml)	13.00 ± 0.64	15.36 ± 0.71	18.04 ± 1.21	16.05 ± 1.01	**0.012** 0.086 **0.001**	**0.001** **0.048** 0.262 1.000
Visfatin (ng/ml)	3.97 ± 0.44	3.94 ± 0.23	4.05 ± 0.26	4.75 ± 0.44	0.418 0.281 0.440	1.000 0.862 1.000 0.946
Adiponectin (μg/ml)	5.50 ± 0.38	4.50 ± 0.37	4.23 ± 0.75	3.79 ± 0.30	0.061 0.676 **0.016**	0.291 **0.012** 1.000 1.000
C27	3.81 ± 0.44	6.90 ± 1.30	5.89 ± 0.92	7.10 ± 0.80	**0.033** 0.323 **0.010**	0.570 **0.011** 1.000 1.000

*Data are presented as mean ± SEM. One-way ANOVA and post-hoc Bonferroni tests was used for the comparison of various clinical and biochemical parameters tested. n = 277. TC, Total Cholesterol; HDL, high density lipoprotein; LDL, low density lipoprotein; and TGL, triglycerides; FBG, fasting blood glucose; HbA1c, hemoglobin A1c; PAI-1, Plasminogen activator inhibitor-1. Bold values represent significance*.

### DNAJC27 expression in circulation

**Figure 1:**
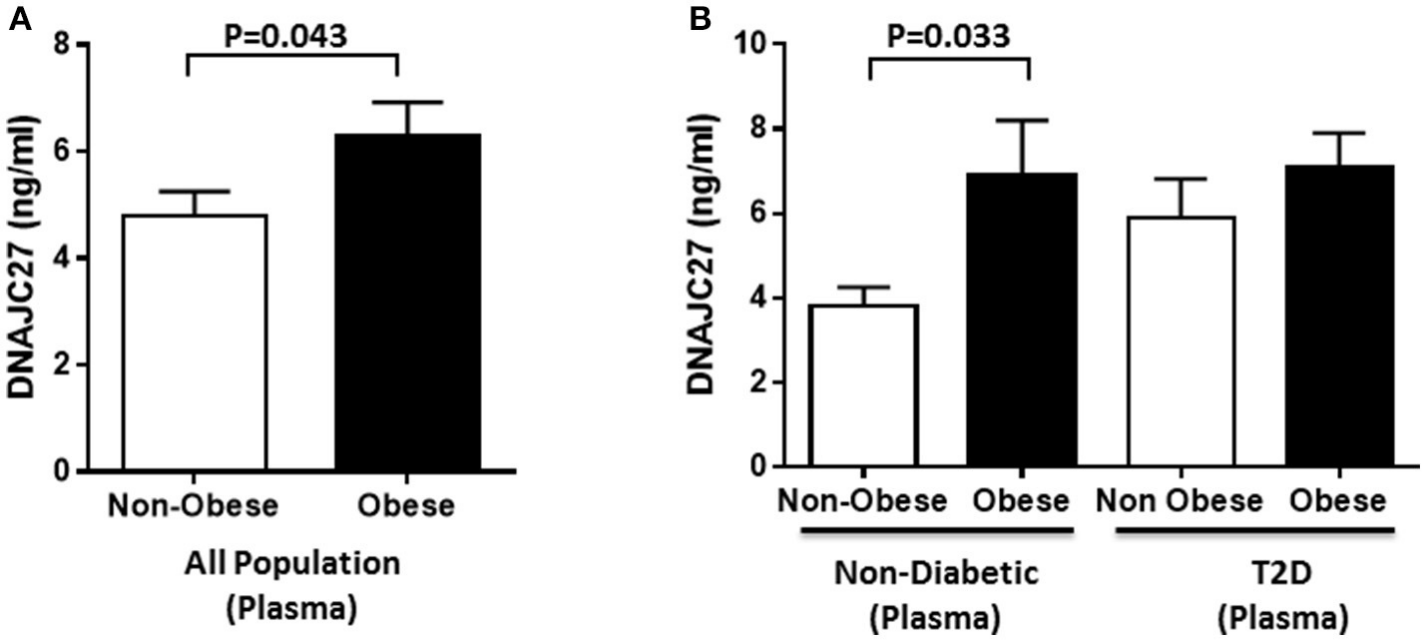
DNAJC27 Level in plasma comparing obese to non-obese individuals. **(A)** Plasma level of DNAJC27 in all population (*n* = 277). **(B)** Plasma level of DNAJC27 in non-diabetic (*n* = 160) and in T2D (*n* = 117).

In the population under study, the level of DNAJC27 in plasma was significantly higher in obese (6.28 ± 0.64 ng/mL) than in non-obese individuals (4.80 ± 0.45 ng/mL) with *P* = 0.043 (Figure 1A). When the population was stratified on the basis of diabetes, within the non-diabetic population a significant increase was observed in the plasma level of DNAJC27 in obese (6.90 ± 1.30 ng/mL) compared with non-obese individuals (3.81 ± 0.44 ng/mL; *P* = 0.033) However, among the people with T2D there was no significant difference in the DNAJC27 level between obese and non-obese (Figure 1B).

When the whole population was classified on the basis of their diabetic status. There was a significant increase in the plasma DNAJC27 level among the T2D individuals (6.46 ± 0.6 ng/mL) as compared to non-diabetic individuals (4.77 ± 0.53 ng/mL) with *P* = 0.037 (Supplementary Figure 1A).

### Obesity and T2D biomarkers in circulation

Plasma level of leptin was significantly higher in obese individuals (9.61 ± 0.62 ng/mL) than in non-obese individuals (5.43 ± 0.36 ng/mL) with *P* < 0.0001. Insulin level was also higher in obese individuals (28.2 ± 3.23 U/L) than in non-obese individuals (17.33 ± 1.82 U/L) with *P* = 0.004 (Table 1). When the study participants were classified based on diabetes, plasma level of Leptin was significantly higher in obese non-diabetic individuals (10.11 ± 0.95 ng/mL) than in non-obese non-diabetic individuals (5.31 ± 0.44 ng/mL) with *P* < 0.0001. Also, the plasma insulin level was significantly higher in obese non-diabetic individuals (30.82 ± 4.81 U/L) than in non-obese non-diabetic individuals (15.86 ± 2.30 U/L) with *P* = 0.001. Plasma level of PAI-1 was significantly higher in non-diabetic obese individuals (15.36 ± 0.71 ng/mL) compared with that in non-diabetic non-obese individuals (13.00 ± 0.64 ng/mL) with *P* = 0.012 (Table 2). Among the diabetic population, only the plasma level of Leptin was significantly higher in obese (9.03 ± 0.77 ng/mL) when compared with non-obese individuals (5.68 ± 0.65 ng/mL) with *P* = 0.004 (Table 2).

### DNAJC27 expression in PBMCs and adipose tissue

**Figure 2:**
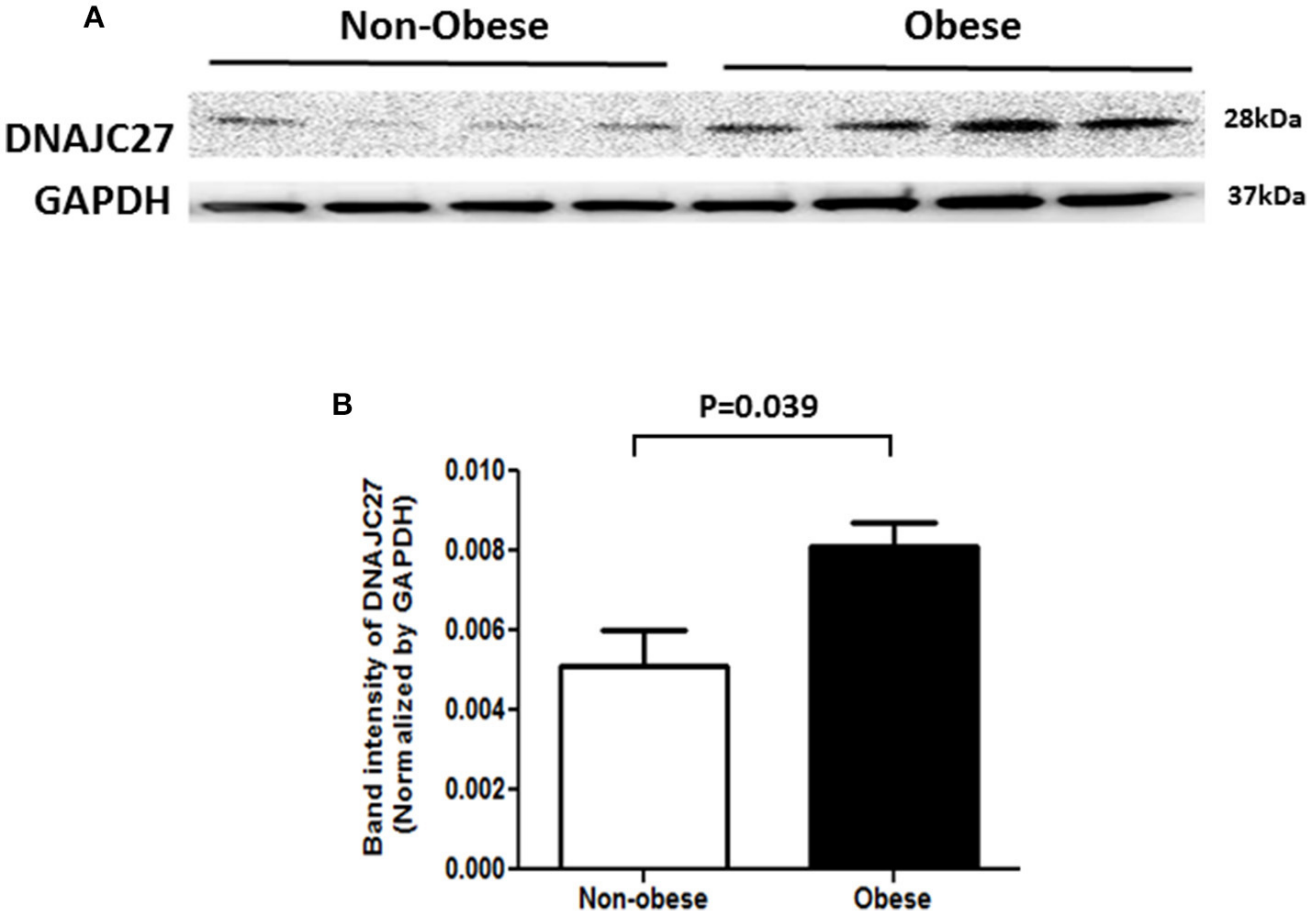
Western Blot analysis of protein from PBMCs of Non-obese and obese individuals that were Non-diabetic. (*n* = 4 each group). **(A)** Western blot image. **(B)** Band intensity quantified after normalizing with GAPDH.

In order to confirm our previous findings from proteomics analysis on DNAJC27 protein expression (20), we performed western blot analysis on proteins extracted from PBMCs of non-obese and obese subjects (*n* = 4 for each group). As shown in Figure 2, a significant increase was observed in DNAJC27 protein expression in obese participants compared with that in non-obese subjects within the non-diabetic population set.

**Figure 3:**
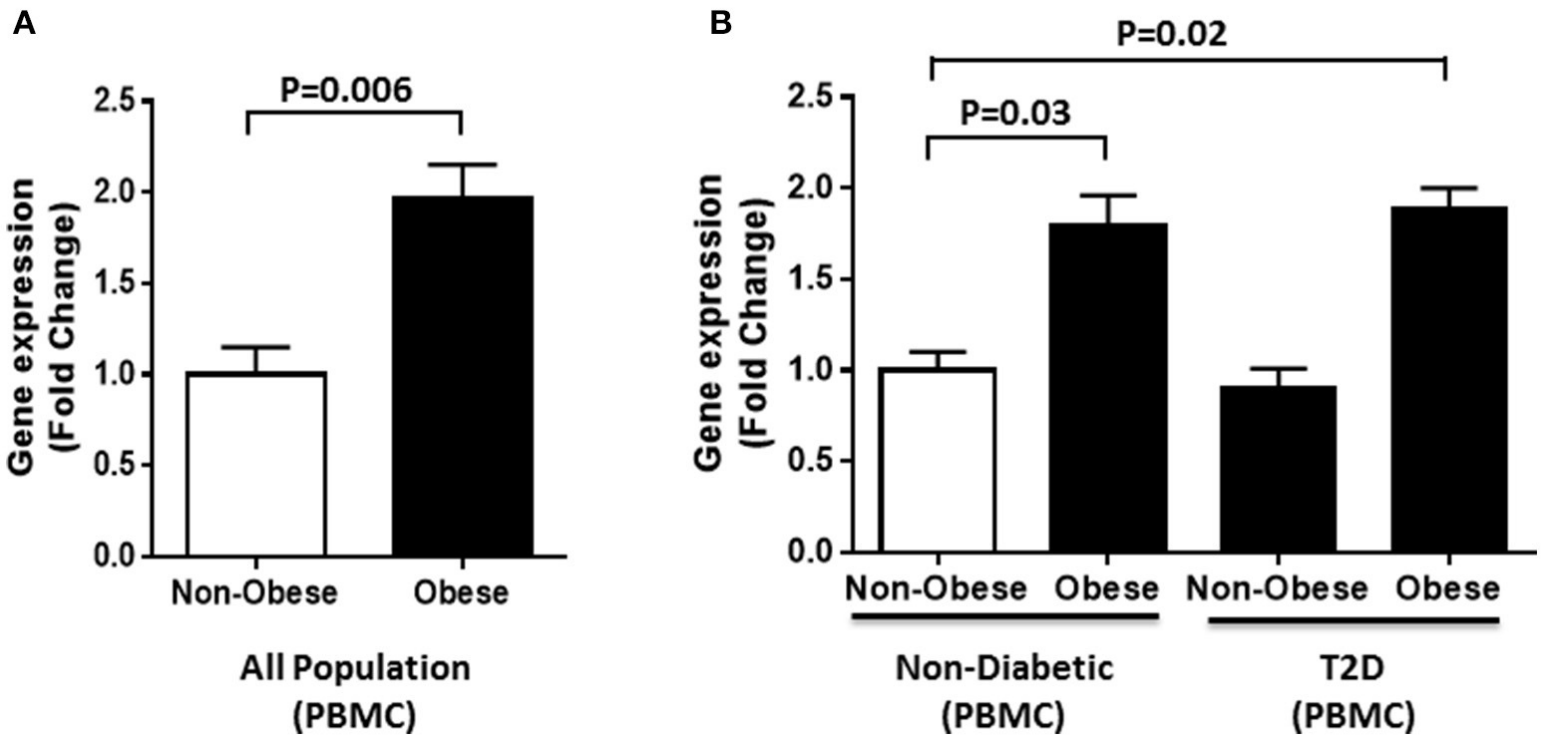
DNAJC27 expression in PBMC comparing obese to non-obese individuals. **(A)** Expression of DNAJC27 in all population (*n* = 60). **(B)** Expression of DNAJC27 in non-diabetic (*n* = 30) and in T2D (*n* = 30).

Furthermore, gene expression analysis was performed using RNA from PBMCs of obese and non-obese individuals selected from the whole population (*n* = 60). In agreement with the circulation data, our results showed a significant increase in DNAJC27 gene expression in obese individuals compared with non-obese (1.97-fold) with *P* = *0.006* (Figure 3A). We also categorized our population based on their diabetic status. Within the non-diabetic sub population (*n* = 30), we observed a significant increase in the gene expression of DNAJC27 in obese (*n* = 15) as compared to non-obese (*n* = 15) individuals (1.8-fold) with *P* = *0.03*. Similarly, among the T2D individuals (*n* = 30), the obese individuals (*n* = 15) showed a higher DNAJC27 expression as compared to non-obese (*n* = 15) (1.72-fold) with *P* = *0.042*.When we compared Obese T2D individuals to non-obese non-diabetic individuals we observed a significant increase in gene expression of DNAJC27 (1.89-fold) with *P* = 0.02. However, no significant difference was observed between non-obese non-diabetic and non-obese T2D individuals (Figure 3B).

**Figure 4:**
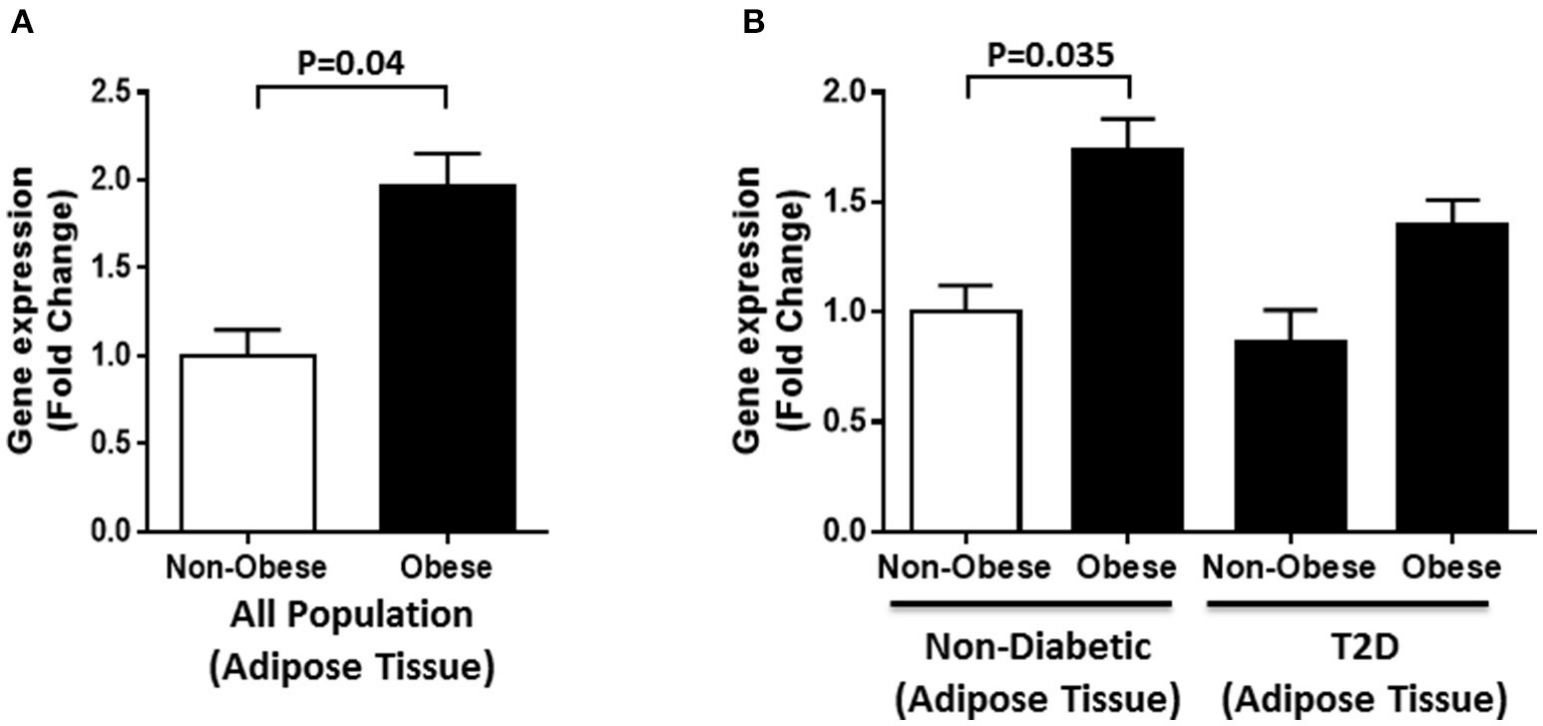
DNAJC27 expression in adipose tissue comparing obese to non-obese individuals**. (A)** Expression of DNAJC27 in all population (*n* = 40). **(B)** Expression of DNAJC27 in non-diabetic (*n* = 20) and in T2D (*n* = 20).

Gene expression analysis was also performed on adipose tissues from obese and non-obese individuals selected from the whole population (*n* = 40). A significant increase in DNAJC27 expression was seen in obese individuals compared with that in non-obese individuals (1.7-fold) with *P* = 0.04 (Figure 4A). We further evaluated RNA expression levels in obese and non-obese individuals with T2D and without diabetes (*n* = 10 for each group). Within the non-diabetic population, we found a significant increase in DNAJC27 expression in the obese when compared to non-obese individuals (1.74-fold) with *P* = 0.035. No significant increase was seen in the T2D population between obese and non-obese individuals. Similarly no significant increase was observed when we compared obese and non-Obese T2D individuals to non-obese non-diabetic individuals (Figure 4B).

We classified the whole population on the basis of their diabetic status. We observed that there was a significant increase in the DNAJC27 gene expression in PBMC (1.8-fold) with *P* = 0.044 (Supplementary Figure 1B) and adipose tissue (1.6-fold) with *P* = 0.048 in the diabetic individuals when compared with non-diabetic individuals (Supplementary Figure 1C).

### Correlation analysis

We performed correlation analysis to evaluate the association of DNAJC27 level with various clinical and biochemical parameters, and to understand the consequence of its dysregulated expression in our study sample. Correlation analysis was done only on the non-diabetic subset of the whole population under study adjusted for age. As shown in Table 3, among all the clinical parameters, significant positive correlation was observed between DNAJC27 level and FBG level (*r* = 0.316, *P* = 0.009). There was a strong positive correlation of DNAJC27 level with HOMA-IR (*r* = 0.316, *P* = 0.01). A positive correlation was also observed between TGL level and DNAJC27 (*r* = 0.215, *P* = 0.05).

**Table 3:** Correlation between circulating DNAJC27 protein and physical, clinical, and biochemical parameters in the non-diabetic population adjusted for age.

**(*n* = 160)**	***r***	***P-value***
BMI (Kg/m2)	0.176	0.085
TC (mmol/L)	0.074	0.293
HDL (mmol/L)	0.023	0.434
LDL (mmol/L)	0.049	0.360
TGL (mmol/L)	0.215	**0.05**
FBG (mmol/L)	0.316	**0.009**
HBA1C (%)	0.078	0.288
Insulin (U/L)	0.026	0.425
HOMA-IR	0.316	**0.01**
Leptin (ng/ml)	0.600	**<0.0001**
Resistin (ng/ml)	0.466	**0.002**
PAI-1 (ng/ml)	0.236	0.083
Visfatin (ng/ml)	0.055	0.374
Adiponectin (μg/ml)	0.080	0.282

*DNAJC27, DnaJ heat shock protein family (Hsp40) member C27. The correlation was assessed by Spearman's rank correlation, it was based on plasma level of DNAJC27 obtained by ELISA method and various physical and clinical parameters. Bold values represent significance*.

Interestingly, the correlation analysis of DNAJC27 with the biochemical markers (Table 3) showed a strong positive association with leptin (*r* = 0.600, *P* < 0.0001) and resistin (*r* = 0.466, *P* = 0.002). A trend of positive correlation with BMI and a trend of negative correlation with PAI-1 was also observed. No significant correlation was observed with the other parameters.

DNAJC27 level in plasma was correlated to its expression level in adipose tissue and PBMC (expressed as ΔΔCT). In our population, the level of DNAJC27 in plasma shows an inverse correlation with the ΔΔCT expression (and hence a positive correlation with fold change) in PBMC (*r* = −0.381; *P* = 0.003) and adipose tissue (*r* = −0.574; *P* < 0.001). However, there was a strong positive correlation between expression of DNAJC27 in PBMC and adipose tissue (*r* = 0.777; *P* < 0.001) (Supplementary Figure 2).

## Discussion

This study was designed in continuation with our research on identifying members of the HSP40 family that may be differentially expressed in individuals with obesity and T2D. To the best of our knowledge, this is the first report to note that DNAJC27 is upregulated in individuals with obesity, both in circulation and adipose tissues. This finding is particularly interesting because our previous studies on other members of the same HSP40 family, namely DNAJB3, DNAJB5, and DNAJB7 showed a decrease in their expression levels in individuals with obesity and T2D. We investigated the role of DNAJB3 in insulin signaling and proposed a model to explain the possible mode of action of DNAJB3. We suggested that DNAJB3 could modulate the insulin signaling pathway by improving insulin acquisition and glucose uptake, which are tightly associated with the reduction of JNK phosphorylation and an increase in AKT and AS160 phosphorylation (19).

Little is known regarding the role of DNAJC27 in obesity and T2D. DNAJC27, which is also known as RBJ, RJL, or RABJS has been identified as a member of the sixth subfamily of Ras-related small GTPases and is characterized by the fusion of the small GTPase domain with the HSP70-interacting J domain (25, 26). Recently, DNAJC27 (RBJ) has been identified as a nuclear protein that can promote carcinogenesis and tumor progression via nuclear accumulation of mitogen-activated protein/extracellular signal- regulated kinase (ERK) kinase (MEK) 1/MEK2 and activation of ERK1/ERK2 (27). ERK is one of the components of the MAPK pathway, which is involved in different biological processes such as cell proliferation and differentiation, metabolism, response to stress, and inflammation (28). The MAPK pathway is known to play a role in the pathophysiology of obesity and T2D. In adipocytes the MAPK pathway has been shown to be involved in regulating the differentiation and proliferation of cells (29). It has been reported that extracellular signal-regulated kinase (ERK) activation enhances early stages of adipogenesis (30). Similarly, basal activation of ERK was found to be increased in diabetic human and rodent adipose tissue (31). Knockout studies in mice have shown that ERK1 is required for adipogenesis both *in vitro* and *in vivo*. Mice lacking ERK1 were resistant to obesity and protected from insulin resistance when subjected to a high fat diet (32). It is also reported that metabolic and inflammatory stresses associated with obesity and T2D, increase the activity of JNK and ERK in several tissues. Specifically, it was shown that ERK pathway mediates the downregulation of IRS1 expression induced by inflammatory cytokines (33). Knowing that DNAJC27 is involved in the activation of ERK in cancer (27), it is possible that it plays a role through the MAPK pathway affecting stress response and inflammation which in turn plays a role in the pathogenesis of obesity.

We also evaluated the plasma levels of different obesity and T2D markers such as adiponectin, leptin, resistin, visfatin, PAI-1, and insulin. As previously identified, our data also showed an increase in leptin level in the circulation of obese individuals (34–36). As expected, obese individuals showed an increase in the circulation level of insulin. Though the insulin levels were observed to be significantly different between obese and non-obese individuals, in our non-diabetic population, plasma C-peptide level showed no difference. Insulin and C-peptide are secreted from the β cell in comparable molar concentrations and significant association between them is expected. The fact that both molecules have different half-life and metabolic clearance rates render the correlation between them less apparent. The half-life of C-peptide is longer than insulin (20–30 and 3–5 min, respectively) (37). Within the non-diabetic population, there was an increase in PAI-1 level in obese individuals. Our data agrees with previously reported studies on dysregulation of different adipokines expression associated with obesity.

Furthermore, we performed correlation analysis for evaluating the association of the levels of various markers with the DNAJC27 level in circulation. Interestingly, we found that DNAJC27 had a positive association with leptin, which is one of the major adipokines strongly associated with obesity. Leptin is a hormone that is produced by the adipocytes and plays a pivotal role in maintaining energy homeostasis. It was long thought that leptin protects the body from increased storage of body fat. However, several studies performed on animal models and humans led to the conclusion that diet-induced obesity may result in leptin resistance (38–40). Little is known regarding the mechanism that leads to leptin resistance in obesity. Nonetheless, research has suggested several mechanisms that may be involved in the development of leptin resistance, including defective leptin transport across the blood–brain barrier (41); the attenuation of leptin signaling (42, 43); deficiency and variations in the *LEP* and *LEPR* genes (38, 44, 45); ER stress (46–48); and inflammation (49). It is therefore possible that DNAJC27 and leptin are interconnected through one or more of the above-mentioned mechanisms. Elucidating the relationship between these two proteins is an objective of our follow-up studies.

A strong association was also observed between DNAJC27 level and resistin, another adipokine implicated in the etiology of obesity. Results from human studies addressing the level of resistin in obesity and T2D have varied, from showing an increase to no change (50). Nonetheless, it has been shown through gene expression studies in humans that resistin is predominantly expressed in PBMCs, macrophages, and the bone marrow (51). Therefore, resistin may play a more important role in inflammatory processes rather than in processes involved in adiposity and glucose homeostasis (52).The positive association of DNAJC27 with leptin and resistin supports possible pro-inflammatory roles of this protein in obesity and T2D, in contrast with those of other proteins in the same family, such as DNAJB3.

Previous genome-wide studies performed on Asian and European sub-populations showed an association of increasing BMI with DNAJC27 gene loci (21, 53, 54). In the European study, DNAJC27 was found to be primarily associated with glucose-related traits and insulin-related phenotypes (53). This relationship was confirmed in our association studies, where we observed a significant positive association of DNAJC27 with HOMA-IR as well as with FBG. Further research is required to understand the mechanism by which DNAJC27 is involved in the manifestation of these traits since this study was limited by its cross-sectional design. However, we studied the expression level of DNAJC27 in circulation as well as in adipose tissue and PBMC which confirmed DNAJC27 level is increased with obesity. Our results showed parallel changes between systemic (plasma) and endogenous (PBMC and adipose tissues) levels of DNAJC27 expressions which supports the notion that PBMC and adipose tissues can act as possible source of the circulating DNAJC27 protein.

In conclusion, our findings shed light on yet another member of the HSP40 protein family, DNAJC27. Unlike DNAJB3, this protein was found to be increased in obesity. The difference in the DNAJC27 expression level and its association with various markers of obesity suggests the possibility that this protein plays a role in the dysregulation of stress responses, inflammatory signaling, and glucose metabolism. Given the role of various HSPs in insulin signaling and inflammation, further studies are necessary to establish the mechanism of action of this protein and its functional role in inducing inflammation, and its role in the insulin signaling pathway.

## Author contributions

PC and IA-K designed and performed the experiments, analyzed data, and wrote the manuscript. DS performed data analysis and critically reviewed the results section. AA-E, DA-S, MA, and SAE performed experiments on gene expression. JT critically revised the manuscript. FA-M experimental design. MA-F and JA designed, analyzed experimental data and critically revised the manuscript.

### Conflict of interest statement

The authors declare that the research was conducted in the absence of any commercial or financial relationships that could be construed as a potential conflict of interest. The reviewer LR and handling Editor declared their shared affiliation.

## Supplementary material

Supplementary Figure 1 DNAJC27 level in plasma, PBMCs and adipose tissues comparing non-diabetic and T2D individuals in all population **(A)** Comparing DNAJC27 Level in plasma in all population (*n* = 277). **(B)** Comparing DNAJC27 expression in PBMC in all population (*n* = 277). **(C)** Comparing DNAJC27 expression in adipose tissue in all population (*n* = 277).

Supplementary Figure 2 Correlation between circulating DNAJC27 in plasma and DNAJC27 expression in PBMCs and Adipose Tissue.

Supplementary Table 1 Physical and biochemical characteristics of the whole population categorized based on diabetes.
