# Supplementary figures and images for: Increased Circulation and Adipose Tissue Levels of DNAJC27/RBJ in Obesity and Type 2-Diabetes
Source: Front Endocrinol (Lausanne). 2018 Aug 7;9:423. doi: 10.3389/fendo.2018.00423 (PMC6090877; doi:10.3389/fendo.2018.00423)

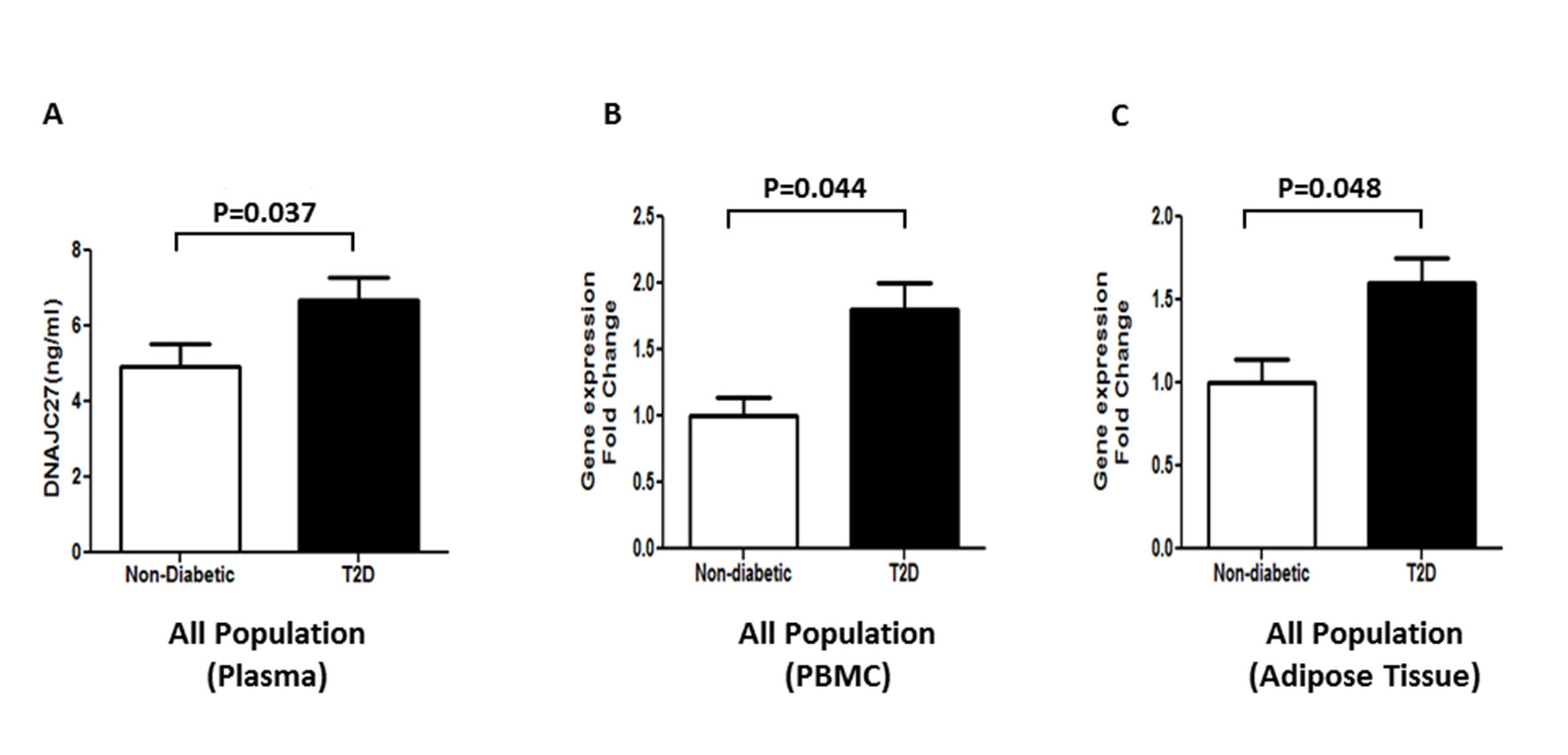

Supplement: Supplementary Figure 1 — DNAJC27 level in plasma, PBMCs and adipose tissues comparing non-diabetic and T2D individuals in all population (A) Comparing DNAJC27 Level in plasma in all population (n = 277). (B) Comparing DNAJC27 expression in PBMC in all population (n = 277). (C) Comparing DNAJC27 expression in adipose tissue in all population (n = 277). [file Image_1.TIF]

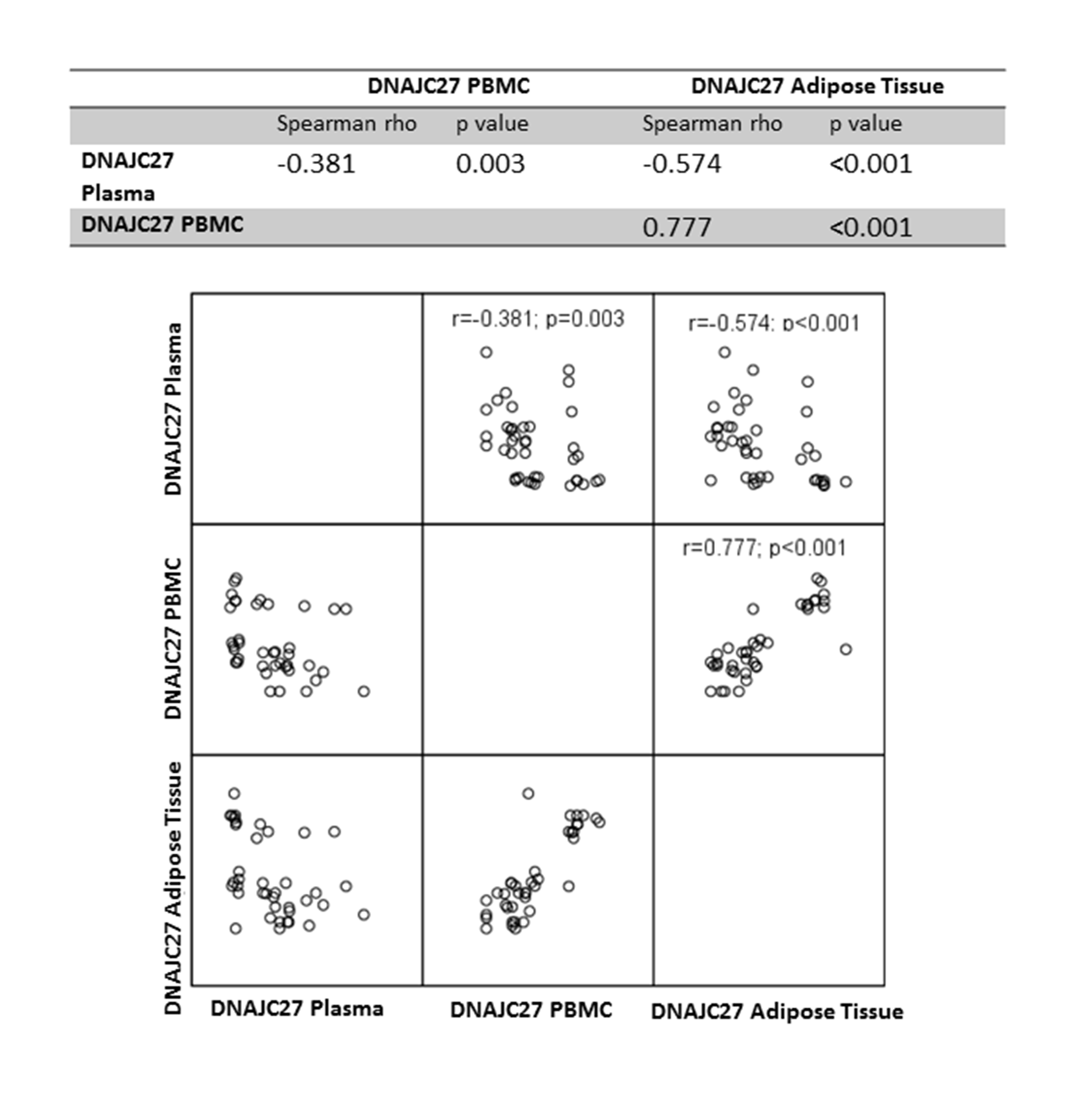

Supplement: Supplementary Figure 2 — Correlation between circulating DNAJC27 in plasma and DNAJC27 expression in PBMCs and Adipose Tissue. [file Image_2.TIF]
