# Supplementary material for: Increased Circulation and Adipose Tissue Levels of DNAJC27/RBJ in Obesity and Type 2-Diabetes
Source: Front Endocrinol (Lausanne). 2018 Aug 7;9:423. doi: 10.3389/fendo.2018.00423 (PMC6090877; doi:10.3389/fendo.2018.00423)
Supplement: Supplementary Table 1 — Physical and biochemical characteristics of the whole population categorized based on diabetes. [file Table_1.DOCX]

**Supplementary Table 1:** Physical and Biochemical characteristics of the whole population categorized based on diabetes.

| All population | non-diabetic | T2D | p-Value |
| --- | --- | --- | --- |
| Age (Years) | 41.86 ± 0.10 | 52.18 ± 0.86 | **<0.001** |
| TC (mmol/L) | 5.13 ± 0.08 | 4.92 ± 0.12 | 0.130 |
| HDL (mmol/L) | 1.33 ± 0.04 | 1.19 ± 0.04 | **0.008** |
| LDL (mmol/L) | 3.24 ± 0.07 | 3.01 ± 0.10 | 0.069 |
| TGL (mmol/L) | 1.23 ± 0.07 | 1.64 ± 0.10 | **0.002** |
| FBG (mmol/L) | 5.35 ± 0.10 | 8.31 ± 0.29 | **<0.001** |
| HBA1C (%) | 5.63 ± 0.07 | 7.77 ± 0.17 | **<0.001** |
| Insulin (U/L) | 22.27 ± 2.52 | 23.78 ± 2.96 | 0.698 |
| C-peptide (pg/ml) | 4.53 ± 0.52 | 3.61 ± 0.49 | 0.200 |
| Leptin (ng/ml) | 7.36 ± 0.53 | 7.60 ± 0.56 | 0.759 |
| Resistin(ng/ml) | 3.38 ± 0.15 | 3.31 ± 0.13 | 0.704 |
| PAI-1 (ng/ml) | 14.01 ± 0.49 | 16.90 ± 0.78 | **0.002** |
| Visfatin (ng/ml) | 3.96 ± 0.27 | 4.45 ± 0.28 | 0.203 |
| Adiponectin (μg/ml) | 5.07 ± 0.27 | 3.95 ± 0.34 | **0.010** |

Data are presented as mean ± SEM. Student-t test was used for the comparison of various clinical and biochemical parameters tested. n=277. BMI (Body Mass Index); TC (Total Cholesterol); HDL (high density lipoprotein); LDL (low density lipoprotein) and TGL (triglycerides); FBG (fasting blood glucose); HbA1c **(haemoglobin A1c); PAI-1 (**Plasminogen activator inhibitor-1)
